# Translational actomyosin research: fundamental insights and applications hand in hand

**DOI:** 10.1007/s10974-012-9298-5

**Published:** 2012-05-26

**Authors:** Alf Månsson

**Affiliations:** School of Natural Sciences, Linnaeus University, 39182 Kalmar, Sweden

**Keywords:** In vitro motility assay, Heavy meromyosin, Nanotechnology, Lab-on-a-chip, Molecular motors

## Abstract

This review describes the development towards actomyosin based nanodevices taking a starting point in pioneering studies in the 1990s based on conventional in vitro motility assays. References are given to parallel developments using the kinesin–microtubule motor system. The early developments focused on achieving cargo-transportation using actin filaments as cargo-loaded shuttles propelled by surface-adsorbed heavy meromyosin along micro- and nanofabricated channels. These efforts prompted extensive studies of surface–motor interactions contributing with new insights of general relevance in surface and colloid chemistry. As a result of these early efforts, a range of complex devices have now emerged, spanning applications in medical diagnostics, biocomputation and formation of complex nanostructures by self-organization. In addition to giving a comprehensive account of the developments towards real-world applications an important goal of the present review is to demonstrate important connections between the applied studies and fundamental biophysical studies of actomyosin and muscle function. Thus the manipulation of the motor proteins towards applications has resulted in new insights into methodological aspects of the in vitro motiliy assay. Other developments have advanced the understanding of the dynamic materials properties of actin filaments.

## Introduction

Molecular motors transport and sort cargoes in cells and underlie both cell-, and organism-motility. As such they are key effectors in the running of cellular fabrication and analysis systems, e.g., those requiring transportation of building blocks and signalling molecules between the nucleus and the cell periphery (Hirokawa et al. 2010). Additionally the molecular motors are key effectors in biological self-organization from cellular to societal levels. The growing interest to exploit molecular motor driven systems and cytoskeletal filament components in various applications is therefore not surprising. The efforts towards applications (Agarwal and Hess 2010; Bakewell and Nicolau 2007; Goel and Vogel 2008; Hess 2011; Korten et al. 2010; Månsson et al. 2005; van den Heuvel and Dekker 2007) encompass materials science developments, e.g., active materials and a range of applications in nanoscience such as self-organized pattern generation, biocomputation and development of lab-on-a-chip devices (e.g., for medical diagnostics). In the latter type of devices, separation, detection and readout are achieved on a single micro and/or nanostructured chip.

The basis for the developments in the mentioned areas has generally been the gliding in vitro motility assay (Kron and Spudich 1986; Kron et al. 1991). In this assay, myosin or kinesin motors are immobilized on surfaces and their propulsion of fluorescence labelled actin filaments and microtubules, respectively are observed under different conditions. The developments towards motor driven applications in nanotechnology started with largely explorative studies. In this early work, kinesin 1 propelled microtubules (Dennis et al. 1999; Hess et al. 2001, 2002b; Hiratsuka et al. 2001) and myosin II (or rather heavy meromyosin; HMM) driven actin filaments (Mahanivong et al. 2002; Nicolau et al. 1999; Suzuki et al. 1995, 1997) were guided along micro-, or nanopatterned artificial tracks. These studies, e.g., the pivotal paper (Hess et al. 2001) also demonstrated the potential to use motor propelled cytoskeletal filaments as shuttles for transportation of nano-scale cargoes in various applications (see also Suzuki et al. 1996). The use of the filaments as shuttles differs from intracellular transport where the cargoes are instead attached to the motors walking along the cytoskeletal tracks. The cellular paradigm for cargo transportation has been tested in some in vitro studies with surface adsorbed microtubules (Bohm et al. 2001; Brown and Hancock 2002; Turner et al. 1995) and some studies have described the production of oriented actin filament tracks (Huang et al. 2006; Interliggi et al. 2007). However, the filament shuttle approach has advantages of increased cargo-carrying capacity and more straightforward control of the sliding direction by guiding along micro- and nanofabricated tracks where active motors have been selectively adsorbed. This guiding has later been perfected in systematic studies using both kinesin propelled microtubules (Clemmens et al. 2003a, b; Hiratsuka et al. 2001; Moorjani et al. 2003) and myosin propelled actin filaments (Bunk et al. 2003, 2005b; Mahanivong et al. 2002; Manandhar et al. 2005; Sundberg et al. 2006a, b). Recent studies have also been performed where actin filament bundles rather than isolated actin filaments have been used as shuttles (Takatsuki et al. 2010, 2011).

Surface–motor interaction-mechanisms that prevent motility on certain surfaces and that give optimized function on others have been of key importance in the development of nano- and micropatterned surfaces for guiding of motor propelled filaments. Systematic studies with the goal to understand the motor–surface interactions have therefore been performed both for the microtubule–kinesin system (Fischer and Hess 2007; Hiratsuka et al. 2001; Kerssemakers et al. 2006; Ozeki et al. 2009) and for actomyosin (Albet-Torres et al. 2007, 2010; Balaz et al. 2007; Jaber et al. 2003; Månsson et al. 2008; Nicolau et al. 2007; Persson et al. 2010; Sundberg et al. 2003, 2006a).

An important goal for exploitation of molecular motors is to achieve motor driven lab-on-a-chip systems. Whereas such systems are among the most challenging motor driven devices, they are also closest to the market and, recently, actual proof-of-principle systems have been described (Fischer et al. 2009; Lin et al. 2008). In a lab-on-a-chip (see further above), separation, detection and readout are achieved on a single chip. In this context molecular motor driven transport may substitute separation processes, now relying on fluid flow in narrow channels (microfluidics/nanofluidics, with bulky accessory equipment) but may also form the basis for innovative detection processes (cf. Korten et al. 2010). In view of world population ageing (Department of Economic and Social Affairs Population Division 2002), there is increasing demand for cheap and effective devices for detection of disease biomarkers for diagnostics and disease stratification (for personalized therapies). Device miniaturization is critical in order to increase portability for use in primary care and in developing countries. However, also high sensitivity, with the capability to detect low levels of analytes (e.g., disease biomarkers) is a key issue as well as multiplexed detection of several biomarkers simultaneously. Several advantages of motor driven lab-on-a-chip devices in these regards will be elaborated on below as well as the preconditions for further development of such devices.

In addition to motor driven lab-on-a-chip systems I will also briefly touch on other lines of development such as exploitation of molecular motor driven systems for biocomputation and self-organization phenomena. The motility based approach towards computation involves (Nicolau et al. 2006) the coding of mathematical problems in (micro/nano) fabricated networks followed by exploration of the network by, e.g., motor propelled filaments. In self-organization, patterns or structures form in complex systems as a result of local interactions of the individual elements when the system is left to itself. Such phenomena may be exploited for formation of unique nanostructures, surface gradients and other patterns.

Microtubule–kinesin systems have largely been ahead of actomyosin based systems on the road towards exploitation. This may partly be a matter of chance, e.g., a greater number of researchers in the microtubule-area happened to focus more strongly on the field in the early 2000s. However, the dominance of microtubule–kinesin can also be attributed to more robust cargo-transportation (Korten et al. 2010) and simpler guiding on micropatterned surfaces (due to high flexural rigidity of microtubules) without the need for nanofabrication. However, actomyosin has certain key advantages such as tenfold higher speed (when using myosin II) and greater potential for miniaturization (due to low flexural rigidity of actin filaments).

I will here review the developments towards actomyosin driven devices particularly those exploiting fast myosin II driven transportation. Indeed, whenever “myosin” or “heavy meromyosin” is mentioned it refers to “myosin II”. The account of developments with the microtubule–kinesin driven system (using conventional kinesin 1) is more limited unless the results are essential for full understanding of the developments of actomyosin based devices. For further details on the microtubule–kinesin system, the interested reader is referred to some quite comprehensive review articles (Agarwal and Hess 2010; Goel and Vogel 2008; Hess 2011; Korten et al. 2010; van den Heuvel and Dekker 2007). In addition to a description of the road towards applications I will also mention implications of the results for fundamental insight into motion generating mechanisms, materials properties of actin filaments and methodological aspects of in vitro motility assays that also may be of relevance in functional studies. Finally, possible future developments will be considered.

## Early developments (mainly before 2005)

In vitro motility assays for studies of actomyosin were developed in the eighties (Kron and Spudich 1986; Kron et al. 1991; Spudich et al. 1985; Toyoshima et al. 1987; Yanagida et al. 1984) with early results of key importance for the understanding of actomyosin function and muscle contraction (Harada et al. 1987a, b, 1990; Prochniewicz and Yanagida 1990; Toyoshima et al. 1987, 1989, 1990; Uyeda et al. 1990). The developments also formed the basis for introduction of optical tweezers in the study of single molecule mechanical properties in the 1990s (Finer et al. 1994; Svoboda and Block 1994). Moreover, these pioneering studies together with some more recent papers (Fraser and Marston 1995; Homsher et al. 1992; Warrick et al. 1993) were of particular importance for the later developments towards motor-driven nanodevices.

In order to exploit in vitro motility assays as a basis for cargo transportation in nanotechnological applications it is essential to control the sliding direction of the actin filaments with nm–μm precision. The earliest studies (Suzuki et al. 1995, 1997), relying on empiric evidence for different protein adsorption on different surface chemistries, demonstrated limitation of HMM propelled actin filament sliding to fluoropolymer-tracks or microlithographically produced polymethyl-methacrylate (PMMA) tracks that were most likely hydrophobic compared to the surrounding glass surface. The motility on polymer surfaces were here of similar quality as on nitrocellulose. Some years later, results were presented (Nicolau et al. 1999), using other types of micro-patterned surfaces providing further support that varied hydrophobicity affects motility quality. However, it was not until about 10 years later that the mechanisms for the effects on surface chemistry on motility were investigated in greater detail. Importantly, however, the mentioned studies laid the ground for further exploration. Subsequent to some studies using kinesin-propelled microtubules, we (Bunk et al. 2003) thus investigated different resist polymers (used for nanofabrication by electron-beam lithography) as substrates in the in vitro motility assay showing that some of them supported actin filament motility after HMM adsorption whereas motility was largely inhibited on others. Surprisingly, poor motility was found on PMMA (in contrast to Suzuki et al. 1997) but good motility on another resist polymer (MRL-6000.1XP). Based on this result, electron-beam lithography was used to produce <200 nm wide channels with PMMA walls, MRL-6000 floors for functional HMM adsorption and surrounding PMMA areas for prevention of motility. After HMM adsorption and other key in vitro motility assay incubation steps, actin filament motility was effectively guided along the nanoscale MRL-6000.1XP tracks with only few events where filaments escaped into the solution. The mechanism underlying the difference in motility quality on PMMA between the studies of Suzuki et al. (1997) and Bunk et al. (2003) were not clear at the time. However, oxygen plasma treatment was employed by Bunk et al., causing introduction of negatively charged groups and hydrophilization of the PMMA surface and it was shown later (Sundberg et al. 2006a) that poor or no motility was observed on a PMMA surface that had been subjected to oxygen plasma treatment whereas high-quality motility was observed on PMMA without such prior treatment.

It was clear that any work towards applications could not rely on nitrocellulose as a substrate for HMM adsorption since it cannot be readily nanopatterned. On the other hand, the motility with polymer resists as HMM adsorbing substrates was less than perfect. Therefore, we (Sundberg et al. 2003, 2006a, b) decided to turn to self-assembled silane monolayers that may be readily nanopatterned and that do not suffer from the batch-to-batch variability and other complexities associated with nitrocellulose surface preparations. Moreover, dichloromethylsilane (Fraser and Marston 1995; Warrick et al. 1993) as well as more complex chlorinated organopolysiloxanes (Sigmacote^®^, Sigma-Aldrich Inc., e.g., Harada et al. 1990) had already been used as surface substrates in motility assays after reacting with surface silanols on glass. However, some variability between experiments and labs were reported. Therefore, in order to eliminate the complexities associated with the siloxane polymers and the risk of polymer formation using dichlorodimethyl silane (with two functional chlorine groups; see further Sundberg et al. 2003) we performed in vitro motility assay studies to compare different silanization procedures. In these early investigations it turned out that SiO_2_ and glass surfaces treated with trimethylchlorosilane were ideal substrates for actomyosin motility and suitable for further development of nanopatterned surfaces for motility control. Indeed, no later results have contradicted this idea (cf. Sundberg et al. 2006a; Albet-Torres et al. 2007, 2010, Persson et al. 2010). When the silanization is performed in routine lab environments (ordinary fume hood without controlled environment as in a glove box) it generally leads to surfaces exhibiting similar contact angles with water droplets (similar wetting behavior) as nitrocellulose (advancing contact angles 70–80°; simply denoted “contact angles” below) but, not unexpectedly, considerably lower root-mean-square roughness (Albet-Torres et al. 2007; Sundberg et al. 2003; based on atomic force microscopy). The contact angle of 70–80° is lower than that of a complete trimethylsilyl monolayer which can be >100° (Fadeev and McCarthy 1999) but it is close to values that are frequently reported in the literature for a range of silanization conditions (summarized in Fadeev and McCarthy 1999). Accordingly, the silanization procedure in our hands has been robust against slightly varying procedures (Albet-Torres et al. 2007, 2010; Persson et al. 2010; Sundberg et al. 2003, 2006a). Thus, provided that care is taken to use dry substrates, water-free solutions and properly cleaned and hydrophilized glass/SiO_2_ surfaces (with exposed silanol groups) the resulting contact angle has been in the range 70–80° and with good actomyosin motility. When using vapour phase silanization of thermally grown SiO_2_ in a fume hood (Sundberg et al. 2006a) a tendency has been seen towards slightly higher contact angles (80–85°) but rinsing in water usually brings the contact angle down below 80°. In our first studies of different silanization procedures (Sundberg et al. 2003), we also observed poor motility for glass and SiO_2_ surfaces with low contact angle (<40°; see further below). In an independent study of motility on various polyelectrolyte substrates at about the same time (Jaber et al. 2003) it was found that not only contact angle but also surface charge is important for motility quality (see further below).

## Developments from 2005 and onwards

### Systematic studies of surface–protein interactions

The studies of interactions between myosin motors and the underlying surfaces in the in vitro motility assay have focused on the proteolytic fragment, heavy meromoyosin whose mode of adsorption on nitrocellulose has also been investigated under certain conditions (Toyoshima 1993). This fragment has advantages for nanotechnological applications compared to smaller myosin motor fragments (such as papain S1 and chymotryptic S1) due to higher actin filament velocity. There are also advantages of HMM compared to full length myosin because the myosin tail (corresponding to the light meromyosin fragment), but not the HMM tail (“subfragment 2”), seems to interact non-specifically with actin filaments to inhibit sliding velocity (Guo and Guilford 2004). Furthermore, with myosin it would probably be more difficult to obtain selective motility on different surface chemistries since it, unlike HMM, appears to adsorb in functional form also to some surfaces conventionally used to block protein adsorption, e.g., those coated with bovine serum albumin (Thedinga et al. 1999). Finally, the actual dimensions of the myosin molecules are similar to those of the track/channels used for guiding of actin filaments with the risk of hindered diffusion of myosin from solution into the channels.

**Fig. 1:**
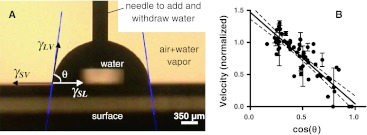
HMM propelled actin filament sliding velocities are correlated with contact angles with water droplets of HMM adsorbing surfaces. **a** The principle for contact angle measurement and the relationship between contact angle θ and surface tensions γ_SV_, γ_LV_ and γ_SL_. For further details, see text. **b** Relationship between sliding velocity (normalized to that on nitrocellulose) and the cosine of the contact angle of the HMM adsorbing surface. Data from Sundberg et al. (2006a), Albet-Torres et al. (2007), and Sundberg et al. (2003)

As mentioned above, there were early indications that the quality (velocity and fraction of motile filaments) of HMM propelled actin filament sliding is correlated with the surface charge (Jaber et al. 2003) and the surface wetting properties (related to hydrophobicity) (Jaber et al. 2003; Nicolau et al. 1999; Sundberg et al. 2003; Suzuki et al. 1997) as quantified by the contact angle of water droplets (Fig. 1). Since a positively charged polylysine surface (Harada et al. 1990) seems to give rather low velocities (mechanisms considered in Albet-Torres et al. 2010) we instead focused on the motility quality on negatively charged surfaces that were made hydrophobic to different degrees.

**Fig. 2:**
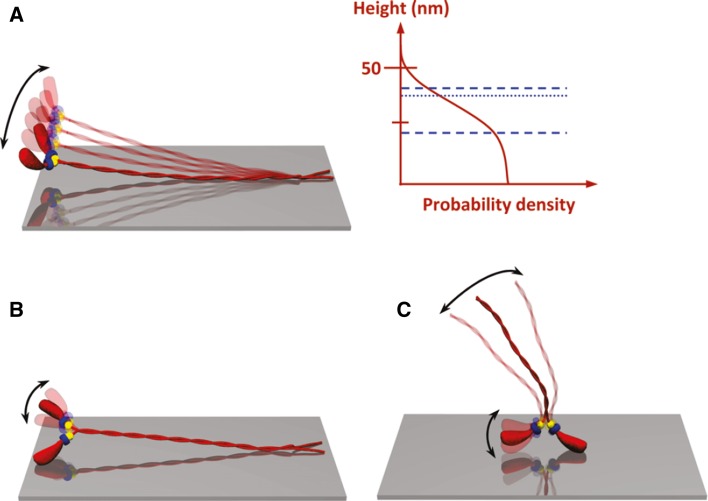
Model illustrating HMM adsorption in different configurations. **a** Configuration with best actin propelling function believed to dominate on moderately hydrophobic surfaces like those derivatized with TMCS. The* diagram *to the* right* illustrates that the HMM molecules in this configuration are likely to be vigorously fluctuating due to thermal motion, creating a distribution of mass with some of the heads reaching >50 nm above the surface in accordance with the equipartition theorem and the stiffness of the coiled-coil subfragment 2 region. The two *horizontal dashed lines* in the *diagram* illustrate HMM layer thicknesses on TMCS derivatized SiO_2_ surfaces on basis of two different interpretations of quartz crystal microbalance (QCM) data. The* horizontal full line* represents the height above the surface of HMM held actin filaments according to fluorescence interference contrast (FLIC) microscopy. **b** Configuration that may be more likely to occur at intermediate contact angles, low temperature (with reduced thermal motion) and low HMM surface densities (see further Månsson et al. 2008). **c** Configurations most likely without HMM binding and HMM propelling capability believed to dominate on negatively charged hydrophilic surfaces. Reprinted, with permission from Persson et al. (2010). Copyright (2010) American Chemical Society. The QCM and FLIC-microscopy based analyses are also from this reference

We found, for surfaces silanized using a range of different monochlorosilanes (Albet-Torres et al. 2007; see also Sundberg et al. 2003, 2006a; Vikhoreva and Månsson 2010), that the sliding velocity (in the presence of viscosity enhancing methylcellulose) increased nearly linearly with contact angle of water droplets on the surface. If sliding velocity is instead plotted against the cosine of the contact angle (Fig. 1) the velocity can be more directly related to the surface properties. Thus, according to the Young relationship:

1 $$ \mathop \gamma \nolimits_{SL} = \mathop \gamma \nolimits_{SV} - \mathop \gamma \nolimits_{LV} \cos \theta $$

where $\mathop \gamma \nolimits_{SL} ,\mathop \gamma \nolimits_{SV} ,\mathop \gamma \nolimits_{LV}$ and θ are the solid–liquid surface tension, the solid–vapor surface tension the liquid–vapor surface tension (constant if only water is used) and the contact angle, respectively. The regression line in Fig. 1b can now be used as a standard curve to obtain $\cos \theta$ (equal to the ratio $(\gamma_{SV} - \gamma_{SL})/ \gamma_{LV} )$ from the observed sliding velocity. Of importance for understanding the molecular mechanism underlying the results of Albet-Torres et al. (2007) it should be mentioned that the increase in contact angle in their study was associated with reduced negative surface charge (as indicated by surface zeta potential measurements). On basis of these results and more recent studies of HMM configurations and function on TMCS-derivatized surfaces, pure SiO_2_/glass and differently charged lipid bilayers, a model for HMM adsorption emerges. Whereas this model should certainly be applicable to different silanized surfaces similar to those studied by Albet-Torres et al. (2007) it may also be applicable to some (but not all) other types of surfaces with varying hydrophobicity (see further below). A key element of the model is the idea that the relationship between velocity and contact angle is attributed to different fractions of HMM molecules being adsorbed in different configurations (Fig. 2). (Albet-Torres et al. 2010; Månsson 2010; Månsson et al. 2008; Sundberg et al. 2006a; Vikhoreva and Månsson 2010). In contrast, the total HMM density on the different surfaces did not appear to differ appreciably. This was suggested by both ATPase assays applied to surface adsorbed HMM (Persson et al. 2010; Sundberg et al. 2006a) and by measurements of changes in HMM fluorescence in the incubation solution during incubation of the motility assay flow cell (Persson et al. 2010). In the HMM configuration that propels actin filaments most effectively (Fig. 2a) HMM seems to be adsorbed to the surface only via the conformationally unstable C-terminal region (site of chymotryptic cleavage to obtain HMM from myosin), i.e., a region where the coiled-coil tends to unfold (see Walker and Trinick 1986). As a result of this mode of adsorption, the heads may extend appreciably away from the surface (Albet-Torres et al. 2010; Persson et al. 2010). This configuration is believed to completely dominate on moderately hydrophobic surfaces (TMCS) whereas the available evidence is consistent with HMM adsorption to pure glass/SiO_2_, preferably via positively charged loops in the actin binding region (Albet-Torres et al. 2010; Månsson et al. 2008; Persson et al. 2010). This idea is consistent with high-speed atomic force microscopy images (Ando et al. 2001a; for movies see Ando et al. 2001b) where myosin V is imaged while adsorbed to freshly cleaved mica[a silicate mineral with, generally low contact angle and negative surface charge (Yang et al. 2007)]. Whereas such surface adsorbed heads seem to exhibit catalytic activity (Persson et al. 2010; Sundberg et al. 2006a) the rate of ATP turnover is appreciably reduced for a large fraction of the heads (considerably larger fraction than seen on TMCS-derivatized SiO_2_) (Balaz et al. 2007). Interestingly, the presence of a fraction of heads with low ATP-turnover rate (probably those immobilized on the surface) is reminiscent of the, so called super-relaxed state in muscle. In this state, myosin heads exhibit particularly low ATP turnover when, as postulated, being parked in an ordered helical arrangement on the thick filament backbone (Hooijman et al. 2011; Stewart et al. 2010). In terms of the model summarized in Fig. 2, the actin propelling configuration of HMM (Fig. 2a) becomes increasingly populated with increased contact angle of the surface [associated with reduced negative surface charge (Albet-Torres et al. 2007)] at the expense of configurations where HMM is adsorbed via the head part (Fig. 2b, c). In addition to expected increase in velocity with increased density (Uyeda et al. 1990) of the actin propelling HMM molecules (Fig. 2a) the velocity may also increase as a result of fewer HMM molecules in the configuration in Fig. 2c. This may, for instance, be expected if there are appreciable non-specific interactions between the HMM tail regions and the actin filaments.

The increased sliding velocity for a contact angle up to approximately 80° is broadly consistent with studies by Nicolau et al. (2007) who used glass and various polymers for HMM adsorption and found increased velocity with an increase in contact angle up to approximately 70° but a reduction in velocity for higher contact angles. The relationship between velocity and contact angle was however, different in a study (Kolli et al. 2010) comparing motility on nitrocellulose (contact angle ~87°), microcontact printed poly(amidoamine) dendrimers (contact angle ~47°) and 3-mercaptopropyl trimethoxysilane (~68°). Here, no motility was found on the silanized surface but similar motility was observed on nitrocellulose and the dendrimer, in spite of widely varying contact angles. This suggests that the relationship between contact angle and velocity on silanized surfaces cannot be universally extrapolated to other surface chemistries. Clearly, several factors are important, out of which surface charge and roughness (Albet-Torres et al. 2007) (uncorrelated with contact angle but possibly related to polymer formation) have already been pointed out. Particularly for high contact angles (>70°) the effects on velocity show appreciable variability. Whereas Nicolau et al. (2007) found a decrease in velocity for contact angles >70°, Kolli et al. (2010) obtained high-quality motility on nitrocellulose with contact angle >80°. In our studies (unpublished) we have observed reduced velocity in occasional experiments with more extensive silanization and a contact angle beyond 80°. Because the HMM molecules (according to the equipartition theorem) will execute vigorous thermal motion around a surface attachment point in the C-terminal region (see Fig. 2a) the head regions will frequently hit the surface and if this is hydrophobic beyond a certain level, one may expect increased risk of entropically driven unfolding. In this connection, it should also be mentioned that (Jaber et al. 2003) observed lack of motility and actin binding by HMM immobilized on a hydrophobic surface.

### Applications for surface characterization

In addition to suggesting appropriate surface functionalization methods for motility supporting regions in nanotechnological applications the studies mentioned above have led us to use TMCS-derivatized surfaces also in fundamental biophysical studies (Vikhorev et al. 2008a) due to more consistent and better characterized properties than nitrocellulose. However, of greater potential interest is the direct relationship between surface tension and sliding velocity because it opens for use of HMM propelled actin filament velocity to characterize surface properties. This would be of particular relevance for characterizing narrow nanostructured channels (cf. Fig. 1) where micro-contact angle measurements (Sundberg et al. 2007) are not possible. This adds to and complements ideas for surface topography characterization (Hess et al. 2002a) that has previously been proposed as an application for kinesin propelled microtubules.

### Strategies for guiding of HMM propelled actin filaments on a chip

**Fig. 3:**
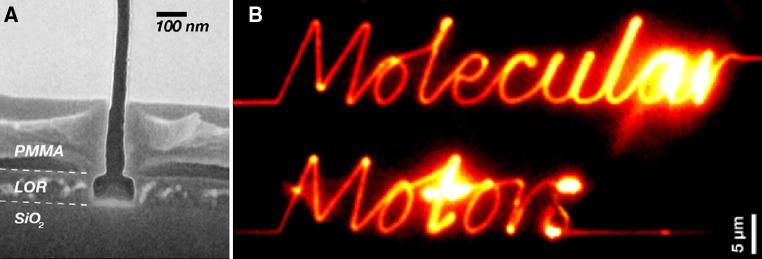
Effective guding of HMM propelled actin filament sliding along combined topographically and chemically defined nanoscale channels. **a** Cross-section of nanoscale channel (Scanning electron microscopy picture) with floor of TMCS-derivatized SiO_2_ and walls of the polymer resists LOR and PMMA (oxygen plasma treated). **b** Fluorescent actin filaments sliding in channels of the type illustrated in A thereby tracing out channel pattern “Molecular Motors”. Image integrated for 6 s. Reproduced from Bunk et al. (2005a) (doi: 10.1088/0957-4484/16/6/014) with permission from the publisher (Institute of Physics)

Guiding of HMM propelled actin filaments to certain regions on a chip along nano- or microscale channels/tracks have been achieved using either topographically (Byun et al. 2009; Mahanivong et al. 2002; Sundberg et al. 2006b) or chemically (Kolli et al. 2010; Manandhar et al. 2005; Nicolau et al. 1999; Sundberg et al. 2006b; Suzuki et al. 1995, 1997) defined track/channels or a combination of these strategies (Bunk et al. 2003, 2005a, b; Byun et al. 2007; Jaber et al. 2003; Sundberg et al. 2006b). One way (Bunk et al. 2005b; Sundberg et al. 2006b) of implementing the combined strategy was by combining hydrophobic/hydrophilic chemical patterning with the formation of effective topographical barriers in the form of partly roofed channels (“inverted T-channels”; Fig. 3a). This was achieved by first opening up the channels by electron-beam-lithography in a PMMA/LOR (lift-off-resist) layer followed by etching of LOR to create a PMMA overhang and treatment with oxygen plasma to make PMMA negatively charged and hydrophilic. Subsequently the channel floors were made motility supporting by vapour phase deposition of TMCS. Channels of this type were found to perform exceedingly well with motility of similar quality as on non-patterned TMCS surfaces, with the motility limited only to the tracks (Fig. 3b) and with virtually no filaments escaping into solution.

**Fig. 4:**
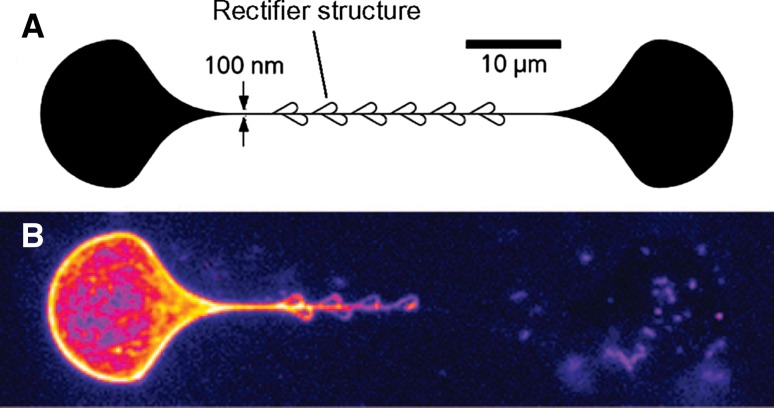
Self-organization of HMM propelled actin filaments on chemically and topographically nanopatterned surface as in Fig. 3. **a** Design of the nanopatterned surface. **b** Self-organization of actin filaments illustrated by integrating several subsequent images at 0.2 s intervals. Brighter color corresponds to brighter fluorescence intensity. The actin filaments were initially immobilized with similar probability to HMM molecules on each of the pear-shaped loading zones. One minute after the addition of ATP, the HMM-induced sliding of the filaments organized them into the illustrated pattern. Reprinted with permission, from Vikhorev et al. (2008b). Copyright (2008) American Chemical Society

In order to ensure unidirectional motion along the nano-scale channels special rectifier structures could be used (cf. Fig. 4; Hiratsuka et al. 2001; van den Heuvel et al. 2005; Vikhorev et al. 2008b). However, theoretical analysis (Sundberg et al. 2006b) has shown that the minimum channel width (*w*_*u*_) for which U-turns are at all possible is given by the following expression:

2 $$ {w_u} = \sqrt {\frac{kT \cdot Lp} {{2d \cdot\rho \cdot  f \cdot {G_{bind}}}}} $$

where *kT* is the Boltzmann factor, *L*_*P*_ is the actin filament persistence length, *d* is the width of a band along the filament (centred on the filament) where myosin heads are available for attachment, f is the duty ratio and *G*_*bind*_ is the average binding energy of the attached myosin heads. This expression suggests (in agreement with experimental results) that channel widths <300 nm would not allow U-turns of HMM propelled actin filaments. The product $n_{heads} = d \cdot \rho \cdot\  f$ in Eq. 2 is the number of attached myosin heads per μm filament length. If *n*_*heads*_ is known, then *G*_*bind*_ can be determined on basis of measurements of *w*_*u*_ because *L*_*P*_ (μm) has been determined for sliding actin filaments (Vikhorev et al. 2008a). This would complement previous measurements obtained under different conditions (Karatzaferi et al. 2004). We (Sundberg et al. 2006b) found *w*_*u*_ = 0.30 μm. By then inserting a value of *L*_*P*_ in the range 7–11 μm (Vikhorev et al. 2008a) (at ionic strength 40 mM) and assuming that *d*, ρ and *f* are in the ranges 26–70 nm (Harris and Warshaw 1993; Uyeda et al. 1990) (4000–13,000 μm^−2^) (Sundberg et al. 2006b), and 0.02–0.06 (Harris and Warshaw 1993; Uyeda et al. 1990) *G*_*bind*_ was estimated to be in the range from 1.4 to 59 kT with a mean value of 19 kT based on midpoint values of the above parameters. Whereas the range based on the extreme parameter estimates is huge (including clearly unrealistic values, e.g., >free energy of ATP turnover [20–25 kT]), the mean value is within the previously determined range of 10–20 kT (Karatzaferi et al. 2004). Obviously, the critical issue is the value of *n*_*heads*_. If this could be measured directly to high precision a considerably better estimate of *G*_*bind*_ would be possible.

The type of channels described above are critical for further developments of actomyosin based nano-devices as further considered below and it was therefore important to know that U-turns are completely prevented for channel widths <300 nm. Moreover, even with channel widths approaching 1 μm, Monte-Carlo simulations of filament paths (Nitta et al. 2006; Nitta et al. 2008) suggest that U-turns are quite unlikely.

### Cargo-transportation using actin filaments as shuttles

Model cargoes, e.g., in the form of the extracellular matrix protein fibronectin (Vikhorev et al. 2008b), fluorescent nanocrystals (Månsson et al. 2004), polystyrene beads (Kaur et al. 2010b; Suzuki et al. 1996), magnetic beads (Kaur et al. 2010a; Marston and Holohan 2005), gold nanoparticles (Patolsky et al. 2004) or liposomes (Takatsuki et al. 2011) have been attached to and transported by actin filaments or (more recently) actin filament bundles (Takatsuki et al. 2010, 2011). The aims of these studies range from new fundamental biophysical insights to nanotechnological applications. Uses in fundamental studies include: nanometer tracking (Kaya and Higuchi 2010) investigations of rotation around the filament long axis (Suzuki et al. 1996) and application of magnetic forces (Marston and Holohan 2005). Often cargoes have been attached to actin via plus-end capping proteins such as gelsolin (Marston and Holohan 2005; Suzuki et al. 1996; Vikhoreva et al. 2008) in order to minimize possible steric clashes with the underlying HMM surface in the case of rotation (Sase et al. 1997) of actin filaments around their long axis. When the purpose has been cargo-transportation in nanotechnology or the formation of metallized actin filaments (possibly with transportation) the cargoes have preferably be attached along the filament for high loading capacity or the formation of an uninterrupted actin based nanowire.

Following the first demonstration of HMM driven transportation of actin filaments with quantum dots attached to the actin monomers via biotin–streptavidin (Månsson et al. 2004) and similar studies for kinesin-propelled microtubules (Bachand et al. 2004), extensive developments have followed with main focus on microtubule–kinesin (Bachand et al. 2004, 2006; Carroll-Portillo et al. 2009a, b; Diez et al. 2003; Hiyama et al. 2009, 2010; Hutchins et al. 2007; Malcos and Hancock 2011; Raab and Hancock 2008; Ramachandran et al. 2006; Rios and Bachand 2009). The considerably fewer numbers of studies with actomyosin in focus may have different reasons (see Introduction) but it is generally believed that it is considerably more difficult to achieve transportation using HMM propelled actin filaments as shuttles. This may both be attributed to: (1) few myosin binding sites per actin filament length compared to the situation for microtubules, (2) the non-processive function and low duty ratio of myosin II motors requiring (also according to point 1) considerably higher motor surface density and more available interaction sites on actin filaments and (3) possibly rotation of the actin filament around its long axis during sliding with the risk of steric clashes with cargoes. On the other hand, strongly related to these challenges (Korten et al. 2010), the HMM propelled actin filament transportation is about ten times faster and the few actin sites are associated with a low diameter and low flexural rigidity opening for more extensive miniaturization. Moreover, one would also expect that cargoes do not have a substantial effect on velocity of HMM propelled actin filaments in contrast to the situation with kinesin propelled microtubules. Thus, in the latter case, the cargoes seem to act as road-blocks (Korten and Diez 2008) for the processive motion of kinesin with temporary stops that will translate into reduced velocity on the motor ensemble level. Similar effects are not expected with non-processive myosin motors unless, of course, motion is generated by mechanisms similar to those proposed, e.g., by Esaki et al. (2007).

In a dominating fraction of the recent studies of cargo-transportation, the cargoes were attached to the microtubules via streptavidin–biotin bonds using covalently biotinylated filaments (e.g., Ramachandran et al. 2006). A similar approach has been used (Vikhorev et al. 2008b) to attach fibronectin to actin filaments whereas the earlier attachment of quantum dots (Månsson et al. 2004) was achieved using biotin–phalloidin. Whereas biotin–streptavidin mediated cargo attachment is rather versatile is has drawbacks such as the risk of formation of inter-filament cross-links unless the cargo-attachment occurs with the filaments immobilized to the motors on the surface (Ramachandran et al. 2006). Such immobilization, on the other hand, has other disadvantages, e.g., problems with routine quality control (e.g., degree of antibody loading) in a future real device without destroying the device. Risks of aggregate formation also exist if homobifunctional crosslinkers (e.g., glutaraldehyde) (Bachand et al. 2006) are used to covalently immobilize antibodies to the cytoskeletal filaments. However, the more recent development of immobilization strategies, using heterobifunctional cross-linkers (Byeon et al. 2010; Grotzky et al. 2011; Iyer et al. 2011), overcomes this obstacle, i.e., it prevents antibody–antibody or actin–actin cross-linking. A version of this approach, allowing attachment of polyclonal antibodies to microtubules has been studied (Carroll-Portillo et al. 2009a) whereas a more versatile method that allows attachment of both monoclonal and polyclonal antibodies was applied quite recently to actin filaments (Kumar et al. 2011, 2012, manuscript submitted). This study demonstrated that several antibody–antigen complexes could be transported at high velocity (~10 μm/s) when attached to HMM propelled actin filaments. Another recent development in cargo-transportation driven by HMM is the use of fascin-bundled actin filaments (Takatsuki et al. 2010, 2011). These bundles are similar to those existing in vivo in filopodia of motile cells. Such bundles seem to have cargo carrying capacity more comparable to microtubules (e.g., transporting liposomes and bacteria) but markedly better than for actin filaments in spite of similar velocity as the latter. In spite of these favourable properties of the bundles, it would also be of interest to further clarify the limitations for cargo-transportation by isolated actin filaments due to their lower complexity and lower flexural rigidity (Claessens et al. 2006; Vikhorev et al. 2008a) of importance for increased miniaturization. In this context it is important to clarify what mechanisms (related to surface charge, hydrophobicity and size of cargo) that are the limiting factors for transportation.

### Towards separation and detection devices

A range of nanostructure-based devices (Giljohann and Mirkin 2009; Lee et al. 2004; Nam et al. 2003; Zhang et al. 2005; Zheng et al. 2005), independent of molecular motors, have been developed to improve bioanalytical systems such as miniaturized biosensors for increased sensitivity, rate of detection and capacity for multiplexing (Giljohann and Mirkin 2009; Jokerst et al. 2010; Ng et al. 2010). These devices are often combined with microfluidics based separation/concentration (Jokerst et al. 2010; Ng et al. 2010; Whitesides 2006) but, whereas the microfluidic chips are indeed small, bulky accessory equipment is required (Jokerst et al. 2010; Whitesides 2006) as well as strong driving forces for liquid transport, at least in nanofluidics (Månsson et al. 2005). It would therefore be of great value if molecular motor driven shuttles could instead drive separation processes and concentrate analytes on detector sites (Fischer et al. 2009; Lin et al. 2008). Thus, making use of the developments of cargo-transportation and guiding along nanosized tracks of filament shuttles it should be possible to transport antibody–antigen cargoes from pick-up zones (Brunner et al. 2007; Sundberg et al. 2006b) to target zones (e.g., a detector) thus substituting microfluidics for separation and concentration purposes. This approach, in addition to avoiding the problems of fluidics mentioned above, would also require truly minimal sample volumes since the analytes to be detected would be transported without simultaneous fluid flow. Indeed, the approach is like affinity based separation with a stationary fluid phase and motile highly miniaturized “solid phase” (the filament shuttle).

**Fig. 5:**
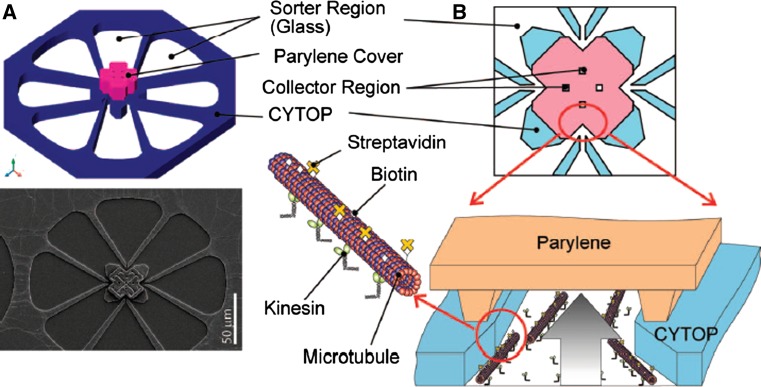
Microstructured device for molecular concentration. After capture of cargoes to be concentrated by microtubules bound to kinesin on the sorter regions, kinesin propels these filaments for concentration in the collector region coated by Parylene. Reprinted and adapted with permission, from Lin et al. (2008). Copyright (2008) American Chemical Society

Proof-of-principle concentration/separation and detection schemes have been described recently using the microtubule–kinesin motor system. In one case (Fig. 5) (Lin et al. 2008) biotinylated microtubules captured fluorescent streptavidin and concentrated it several orders of magnitude with a half-time of tens of minutes. In another device (Fischer et al. 2009) antibodies against glutathione-*S*-transferase (GST) were attached to surface immobilized microtubules on a circular (800 μm wide) pick-up zone using biotin–streptavidin links. This assembly of the final device was followed by addition of analyte (GST). Following binding of GST to the microtubule-bound antibodies it was concentrated at the device edges during a period of hours by means of kinesin driven transport.

Notable for the mentioned proof-of-principle devices is that concentration of the analyte for detection was rather slow compared to existing high-sensitivity methods (Georganopoulou et al. 2005; Mulvaney et al. 2009; Nam et al. 2003; Rissin et al. 2010) and either a simple recognition molecule (biotin) was used or antibodies were attached to the cytoskeletal filaments when immobilized to kinesin (see above). Consequently, challenges remain before a practically useful device is achieved and steps are taken as described below.

Thus, the covalent antibody immobilization on actin filaments via heterobifunctional cross-linkers (Byeon et al. 2010; Grotzky et al. 2011; Iyer et al. 2011) would allow attachment of antibodies to the actin filaments in solution thereby overcoming the limitation related to antibody attachment via biotin–streptavidin on the device surface. Further, by exploiting the high speed of HMM driven actin filaments and their low flexural rigidity (Vikhorev et al. 2008a) we expect that actomyosin-driven concentration of analyte on a target (detector) zone should be possible in seconds which is faster than in recent high-sensitivity methods (Georganopoulou et al. 2005; Nam et al. 2003).

Another challenge that needs to be addressed before arriving at a clinically (or otherwise) useful device is to extend the shelf-life to months or, preferably years. An important finding in this connection is that we recently (Albet-Torres and Månsson 2011) demonstrated long-term storage (months) of fully functional in vitro motility assays with adsorbed HMM, actin filaments and ATP-containing assay solution in a −20 °C freezer. This may be compared to weeks in a refrigerator (Sundberg et al. 2003). A very exciting possibility for further improved shelf-life is to use pharmacologic chaperones similar to that described recently (Radke et al. 2012) to rescue motility assays that have deteriorated as a result of ageing.

Finally, it has been recently shown that complex fluid environments such as blood-plasma and serum may severely affect motility (Albet-Torres et al. 2012, manuscript submitted). Whereas dilution of the sample would overcome the difficulties this is highly undesirable from a sensitivity perspective. Therefore, it is of great interest to develop pre-separation methods that allow exchange of the complex fluid environment for optimized buffers before exposing the cytoskeletal filament shuttles and the molecular motors for the analyte of interest (whether a protein or nucleic acid/oligonucleotide).

### Other nanotechnological and materials science applications

Self-organization of complex systems involves formation of patterns or structures as a result of local interactions of the individual system elements when the system is left to itself. Such phenomena are of critical importance in biology from molecular (e.g., thin or thick filaments of muscle) to societal levels (swarm-like behavior of, e.g., birds) and molecular motor driven motion and force-generation play key roles in these contexts. Not unexpectedly, there have been efforts to exploit self-organization phenomena in nanotechnology for the formation of more or less complex nano-, micro- or even macroscale structures. However, unlike in the biological systems the building-blocks in artificial systems often suffer from heterogeneity with respect to size, shape and intermolecular interaction sites. Therefore, it is of interest to produce hybrid systems were the perfection of biological building blocks (e.g., actin monomers, amyloidogenic peptides) are used to guide self-organization of non-biological molecules or particles. This may be useful for creating structures on different levels of hierarchial organization. Also motor driven transport of cytoskeletal filaments has been exploited in such applications. For instance, stable or meta-stable filament bundles of different shapes and length scales may form (Hess et al. 2005; Idan et al. 2012) in the presence of filament cross-linking molecules as well as DNA–micotubule networks with DNA-fragment bound to the microtubules (Diez et al. 2003). In the absence of cross-linkers the filaments instead either move collectively in self-organized swarms (Butt et al. 2010; Schaller et al. 2010; Vikhorev et al. 2008b) with changing shape from time to time or, under other conditions (Kraikivski et al. 2006), they undergo random diffusion like sliding (Vikhorev et al. 2008b). The latter behaviour can be partly controlled by chemical and topographical micro-, and/or nanopatterns and used to produce actin filament gradients of predictable shapes (Vikhorev et al. 2008b). By then immobilizing the actin filament to myosin heads (by removal of ATP) the filaments can be used as templates for attachment of a range of other molecules [e.g., extracellular matrix proteins (Vikhorev et al. 2008b)] by biotin–streptavidin bonds or via antibodies. This could be relevant in applications such as tissue engineering and cell adhesion studies where varying densities of extracellular matrix proteins are of interest as well as for the production of complex electrical circuits. In the latter case the filaments may be derivatized by gold (Patolsky et al. 2004) to produce conducting nanowires.

In order to predict self-organization phenomena of motor propelled actin filaments on a surface the filament paths may be simulated using a Monte-Carlo approach where each instantaneous update (dφ) in angular sliding direction is drawn from a Gaussian distribution with mean value 0 and standard deviation given by:

3 $$ {\text{SD}}_{{\text{d}}\varphi} = \sqrt {\frac{{{{\text{v}}_{\text{f}}} \cdot {{\Updelta t}}}} {{{{\text{L}}_{\text{P}}}}}} $$

where v_f_ is the sliding velocity and Δ*t* the time interval between successive updates in sliding direction. The guiding at edges such as in nanochannels may also be simulated using the Monte-Carlo approach (Nitta et al. 2006, 2008) but inclusion of interactions between filaments [e.g., important under filament crowding and high motor densities (Butt et al. 2010; Kraikivski et al. 2006; Schaller et al. 2010; Vikhorev et al. 2008b)] will require modifications (Kraikivski et al. 2006) to the simulations compared to previous studies (Månsson et al. 2012; Nitta et al. 2006, 2008). The described simulation method is not only important for predicting motor driven pattern formation but is also important for the optimization of devices for concentration and separation as considered above.

In addition to the above applications in biosensing and self-organization it is also of interest to consider biocomputation based on molecular motor driven filaments and other motile objects. [for further applications, see reviews (Agarwal and Hess 2010; Bakewell and Nicolau 2007; Goel and Vogel 2008; Hess 2011; Korten et al. 2010; Månsson et al. 2005; van den Heuvel and Dekker 2007)]. This motility based approach towards computation involves (Nicolau et al. 2006) the coding of mathematical problems in (micro/nano) fabricated networks followed by, e.g., motor propelled filaments or “self-programmable agents” such as microorganisms. It is argued by Nicolau et al. (2006) that the motion of the agents in the confined geometries may be regarded as a computational process with the potential to compute “any Boolean function by appropriately designing the structures and releasing the agents”. In a computational process using actin filaments, the sliding directions, together with the obstacles, supply the input whereas the output could be the distribution of agents after a certain time or other measurable singnal.

Moreover, a computational process utilizing HMM propelled actin filaments, requires a certain degree of control of transport direction at network nodes and the agents should only be allowed to move forward through the network (Nicolau et al. 2006). Such prevention of U-turns and a strict control of guiding at nodes are possible to achieve using channels of the type described above (cf. Fig. 1). However, for optimal performance at the nodes, it might be necessary to apply also active gating (e.g., using electrical fields (Riveline et al. 1998; van den Heuvel et al. 2006) or other methods (Byun et al. 2011). Additionally, for some computations it is important to monitor the path of individual filaments through the network, something that requires the development of both specific detectors and specific labelling methods for the filaments. Thus, whereas simple versions of motor driven biocomputation devices could be composed simply of appropriately designed networks fabricated using channels as in Fig. 1, more complex versions require both gating and individually addressable detection systems. For this reason, a complete motor driven biocomputation device may be more challenging to develop than lab-on-a-chip devices for separation and concentration of analytes.

### Conclusions and perspectives

Of great interest would be the quite realistic development in the near future of practically useful and commercially viable motor driven lab-on-a-chip systems for biosensing in medical diagnostics, environmental and bioterrorism monitoring etc. Moreover, the developments of self-organized systems deserve further exploration. In these studies it would be of interest not only to exploit wild-type but also engineered biological motors (e.g., Tsiavaliaris et al. 2004) or even artificial molecular motors (Bromley et al. 2009; Feringa 2011; Kuwada et al. 2010). For further development of point-of-care diagnostic devices, it will be important to perform in depth studies of the mechanisms that limit the capability of engineered cytoskeletal filament shuttles to transport various cargoes, from single macromolecules to cells. Other important developments would be new approaches for optimizing (Amrute-Nayak et al. 2010) and stabilizing the protein components in order to increase their shelf-life and methods to ensure the compatibility of motor function with complex fluid environments or alternatively, bypassing this complication by innovative approaches. Additionally, it will be important to select appropriate applications for the different motor system to optimally utilize the high cargo-carrying capacity of microtubule kinesin and the high speed and increased capability of miniaturization associated with myosin II propelled actin filaments. It will also be of interest to build further bridges between fundamental studies and applications. Thus, the manipulation of molecular motors and cytoskeletal filaments for use in applications prompt highly specialized studies that may lead to unique fundamental insights that may not be readily obtained in conventional physiological or biophysical studies. For instance, one problem that has been studied in greater detail for this reason is the material properties (e.g., persistence length) of actin filaments (Nitta et al. 2008; Vikhorev et al. 2008a, b) and microtubules (Nitta and Hess 2005; Nitta et al. 2008; van den Heuvel et al. 2006, 2007) and how these properties may change under different conditions. Also the relationship between the filament persistence length and the winding filament paths in the in vitro motility assay (Duke et al. 1995) have been investigated in some detail as well as the mode of motor adsorption to various surface chemistries. The latter information should be of value when interpreting in vitro motility data obtained in fundamental biophysical studies.
